# Development of Tumor Mutation Burden-Related Prognostic Model and Novel Biomarker Identification in Stomach Adenocarcinoma

**DOI:** 10.3389/fcell.2022.790920

**Published:** 2022-03-23

**Authors:** Min Fu, Yongbiao Huang, Xiaohong Peng, Xiaoyu Li, Na Luo, Wenjun Zhu, Feng Yang, Ziqi Chen, Shengling Ma, Yuanyuan Zhang, Qianxia Li, Guangyuan Hu

**Affiliations:** ^1^ Department of Oncology, Tongji Hospital, Tongji Medical College, Huazhong University of Science and Technology, Wuhan, China; ^2^ Department of Medical Oncology, The First Affiliated Hospital, College of Medicine, Zhejiang University, Hangzhou, China

**Keywords:** bioinformatics, mutation burden, stomach adenocarcinoma, survival, multi-omics, immunity

## Abstract

**Background:** Stomach adenocarcinoma (STAD) is one of the most common tumors. Tumor mutation burden (TMB) has been linked to immunotherapy response. We wanted to see if there was any link between TMB and cancer prognosis.

**Methods:** The Cancer Genome Atlas (TCGA) and the Gene Expression Omnibus (GEO) databases were used to obtain mutation data, gene expression profiles, and clinical data. We looked at the differences in gene expression and immune markers between low and high TMB groups, built an immune prognostic model, and created a dynamic nomograph App that may be used in the clinic. Simultaneously, We ran the immunotherapy prediction and model comparison at the same time. Finally, model gene mutation and copy number variation (CNV) were displayed. The cellular functional experiments were used to investigate the potential role of GLP2R in gastric cancer.

**Results:** Firstly, basic mutation information and differences in immune infiltration in STAD are revealed. Secondly, the prognostic model developed by us has good accuracy, and the corresponding dynamic nomograph Apps online and immunotherapy prediction facilitate clinical transformation. Furthermore, GLP2R knockdown significantly inhibited the proliferation, migration of gastric cancer cells *in vitro*.

**Conclusion:** Our findings imply that TMB plays a significant role in the prognosis of STAD patients from a biological perspective. GLP2R may serve as a potential target for gastric cancer.

## Background

The third most common cancer-related fatality is gastric cancer (GC), among which STAD is the most common type of pathological tissue (Bray et al., 2018). Although many therapeutic ways have been available, the high recurrence rate causes a heavy economic burden in both family and healthcare systems (Mehmedagic et al., 2016; Lee et al., 2017). The cause of STAD is still unclear; many factors, including *H. pylori* infection, smoking, environmental factors, poison contact history, etc. are associated with the occurrence of STAD (Naumann, 2005). Although clinicians have enriched therapeutic choices in recent years, many clinical obstacles are still unresolved. Immunotherapy has been a newly developed method to treat tumors by targeting PD-1, PD-L1, CTLA-4, etc (Kim and Seo, 2018; Powles and Morrison, 2018; Zhang et al., 2018).

First-line clinical trials of immunotherapy in combination with conventional therapy have shown improved clinical benefit and survival in patients with gastric cancer, especially among pretreated patients. This has contributed to the accelerated approval of some checkpoint inhibitors. However, the therapeutic effects of single-drug immune checkpoint inhibitors do not seem to meet expectations. Therefore, new immunotherapy algorithms should develop more efficient predictive biomarkers to distinguish between gastric tumor subsets with different clinical responses (Lazăr et al., 2018; Coutzac et al., 2019; Liang et al., 2021).

Previous papers have pointed out the correlation between immunotherapy response and TMB (Rizvi et al., 2015; He et al., 2019). Gene mutations in tumor tissues may produce new antigens through transcription and translation, thus being recognized and targeted by the immune system (Matsushita et al., 2012; Riaz et al., 2016). Not all mutations produce immunogenicity, and immune cells can only recognize a few mutated tumor antigens (Snyder and Chan, 2015). The more variations the tumor has, the more antigens it forms. Higher TMB has a tendency to generate more neoantigens, enhancing the immunogenicity of tumors and improving the response to immunotherapy in clinical applications (Rizvi et al., 2015). Therefore, TMB can be used as a biomarker to evaluate neoantigen load in tumors.

TMB can be utilized as a biomarker to predict the survival rate of patients with advanced gastric cancer after immunotherapy, which aids doctors in making the best decisions possible (Wang et al., 2019; Kim et al., 2020). In terms of mechanism, TGFB2 may have a role in the epithelial-mesenchymal transition (EMT) and TMB in gastric cancer, making it a possible therapeutic target (Yang et al., 2020). CXCR4 may also influence gastric cancer growth and prognosis by influencing immune infiltration, TMB, cytolytic activity, tumor purity, and treatment sensitivity (Li et al., 2020). In general, the TMB trial in stomach cancer warrants additional investigation.

TCGA has currently mapped the mutations in the human cancer genome, providing a wealth of mutation and expression profile data to researchers all around the world. We used STAD samples from the TCGA database to find differentially expressed genes (DEGs) across the high and low TMB groups, as well as investigate the relationship between immune cell infiltration features and TMB, creating and verifying an immune prognostic model. In the end, a series of functional experiments were carried out on GLP2R.

## Materials and Methods

### Data Acquisition

We downloaded various data of STAD patients from the TCGA data portal (https://tcgadata.nci.nih.gov/tcga/) (Hutter and Zenklusen, 2018), including mutation data, gene expression profiles, and clinical data. The STAD probe matrix file (GSE84433 series matrix) and platform file (GPL6947-13512) were also obtained from the GEO database (Barrett et al., 2007). The patient selection criteria for this study was samples of initial gastric adenocarcinoma after primary surgical resection (patients with missing clinical information were not included). To summarize the mutation data, The mutation data were summarized using the Maftools software package (Mayakonda et al., 2018). The relevant parameters of tumor-specific mutant genes were computed to obtain TMB. STAD samples were separated into high and low TMB groups using the median of TMB as the critical value. Then we looked at the relationship between TMB and survival and other clinical factors. Gene expression data from high and low TMB groups were analyzed using limma package (Ritchie et al., 2015). The fold change >2 and the false discovery rate (FDR) < 0.05 were our screening criterion for DEGs.

### DEGs Enrichment Analysis and Immune Infiltration Assessment

We used GSEA 4.0.3 software to perform Gene Set Enrichment Analysis (GSEA) and reported the first five Gene Ontology (GO) keywords and Kyoto Encyclopedia of Genes and Genomes (KEGG) pathways which enrich most significantly in the high TMB group (compared to the low TMB group) (Subramanian et al., 2005).

The fraction of invading immune cells was computed by applying the CIBERSORT algorithm, and the corresponding violin plot was created. Using the CIBERSORT algorithm with 1,000 permutations and the LM22 signature, an accurate analysis of the immune cells in STAD samples was carried out (Chen et al., 2018).

### Visualization of Mutation Data and ssGSEA Analysis

We performed ssGSEA analysis in TCGA-STAD samples using the GSVA package to determine the immune activity of 23 immune-related genesets, and the correction results ranged between 0 and 1. The heat map (pheatmap R package) and violin plots (ggpubr R package) of the tumor microenvironment were created based on the correlation analysis using the ESTIMATE algorithm between high and low risk group. To clarify the relationship between the risk grouping and immune infiltration, we also used the ssGSEA method to calculate the tumor immune cell infiltration score for each STAD sample in the TCGA database, then used the “Bioconductor Limma” R package to run the differential analysis of immune scoring and immune typing, and finally drew the box plot.

### Construction and Multiple Validations of Immune Prognostic Model

We obtained differentially expressed immune genes by intersecting the list of immune-related genes from the Immunology Database and Analysis Portal (ImmPort Database) (Bhattacharya et al., 2014) with the preceding DEGs. We randomly separated TCGA samples into two groups: a training group and an internal validation group after correlating the expression of differentially immune genes with survival time. Univariate COX analysis (*p* < 0.01) identified survival-related immune genes in the training group, and using the R package glmnet, the least absolute shrinkage and selection operator (LASSO) (Tibshirani, 1997) excluded the high correlation genes to avoid the model from overfitting. Finally, the best prognostic model was created using stepwise multivariate Cox regression analysis.

The developed prognostic model formula was used to determine the patient riskScores for the training, internal validation, total, and external validation (GSE84433) groups. We divided the patients in the training, internal validation, total, and external validation groups into a high-risk group and a low-risk group using the median riskScore of the training group as the threshold, plotting the survival curve and Receiver Operating Characteristic (ROC) curve of the training, internal validation, total, and external validation groups with R x64 3.6.3 software. Ultimately, univariate and multivariate prognosis analyses were done on the entire group (*p* < 0.05) to see if the model’s riskScore may be an independent prognostic factor.

The differences in model gene expression were evaluated between the high and low risk groups, with the ggpubr package drawing model gene boxplots to compute the expression differences. To conduct multiple gene survival analysis for all model genes, we used the PROGgeneV2 online program (Goswami and Nakshatri, 2014) and picked the GSE62254 dataset (Cristescu et al., 2015). We created a dynamic nomograph App online based on the aforesaid prognostic model to aid in the rapid calculation of patient survival rates in clinical practice. In order to demonstrate the application method, we randomly selected a low-risk group sample and a high-risk group sample from the training group, and calculated survival probability by inputting gene expression in the dynamic nomograph App.

### Mutation and CNV of Model Genes

We entered the cBioportal website (Cerami et al., 2012) and selected the study (Stomach Adenocarcinoma TCGA PanCancer data) to download the mutation status of model genes. Finally, we investigated the association between CNV of the model genes and immune cells infiltration level, as well as the correlation between immune cells infiltration level and survival of STAD patients by using the Tumor Immune Estimation Resource (TIMER) database (Li et al., 2017).

### Immunotherapy Benefit Evaluation and Model Comparison

To explore whether the above prognostic model can evaluate the efficacy of immunotherapy for patients, we analyzed a series of immunotherapy biomarkers. We examined the differences between the high and low risk groups by uploading the TCGA sample expression profile into the Tumor immune dysfunction and exclusion (TIDE) database (Jiang et al., 2018) and obtaining TIDE, Microsatellite instability (MSI), Dysfunction, and Exclusion scores for each sample.

Furthermore, 5-years ROC curves were used to evaluate the riskScore’s prognostic prediction efficiency with TIDE and tumor inflammation signature (TIS) scores (Danaher et al., 2018), demonstrating the correctness of the prognostic model we developed.

### Drug Sensitivity Testing

We downloaded transcriptome data from the CellMiner database (https://discover.nci.nih.gov/cellminer/) and FDA-certified drug sensitivity–related data to clarify the influence of model genes in the prognostic model on drug sensitivity and tolerance. To investigate the relationship between gene expression and drug sensitivity, the Pearson correlation test was used. Then, we used the “pRRophetic” R package to draw a box plot and analyze the differences in drug sensitivity between high and low risk groups.

### Cell Culture and siRNA Treatment

Human gastric cancer cell lines (BGC823,MKN45) were cultured in RPMI 1640 medium (HyClone, United States)supplemented with 10% fetal bovine serum (FBS, Gibco, United States). These cells were maintained at 37°C under an atmosphere of 5% CO2. GLP2R siRNA were synthesized by RiboBio (Guangzhou, China) and transfected into cells using Lipofectamine 3,000 (Invitrogen, California, United States) according to the manufacturer’s instructions. The cells were cultured in basic RPMI 1640 medium for 12–16 h before the media was for RPMI 1640 medium containing FBS.

### Quantitative Real-Time PCR

RNA was extracted from gastric cancer cell lines (BGC823,MKN45) interfered with si-GLP2R and NC as control. cDNA was synthesized for real-time PCR adopting SYBR Green qPCR mix (Vazyme, China). The primers are listed as following: GLP2R -Forward: TCC​TGG​AAA​TGT​CTC​TGT​ACC​C; GLP2R—Reverse: GGC​GTT​CTC​TAT​CGT​CTG​CC; GAPDH-Forward:GACCACAGTCCATGCCATCA; GAPDH-reverse:GTCAAAGGTGGAGGAGTGGG.

### Western Blot Analysis

RIPA lysis buffer (Servicebio, China) containing PMSF (Servicebio, China) was used to collect proteins from BGC823 and MKN45 cells. 10% sodium dodecyl sulfate–polyacrylamide gel electrophoresis (SDS-PAGE) was applied in separating protein samples and a polyvinylidene fluoride membrane (PVDF) membrane (Invitrogen, Carlsbad, United States) was used to transfer the separated protein. The membrane was blocked in 5% skim milk at room temperature for 1 h in a shaker, and then incubated with the primary antibody:GLP2R (Abconlal, CAT# A6602, 1:1,000), and GAPDH (Proteintech, CAT# 60004-1-Ig, 1:20,000) at 4°C overnight and subsequent incubation with the secondary antibody for 1 h. Finally, the indicated proteins were visualized by West Pico plus Chemiluminescent Substrate (Thermo Fisher Scientific, United States).

### Cell Proliferation and Colony Formation Assays

After transfection with GLP2R siRNA for 48 h, BGC823 and MKN45 cells were cultured in 96-well plates (3,000 cells/well in 100 µl RPMI 1640 medium). The proliferative capacity of treated cells was detected at 24, 48, 72, 96, and 120 h. 10% Cell Counting Kit-8 (CCK8) reagent (Bio-sharp, Hefei, China) was added to each plate according to the kit instructions, and the OD450 value was analyzed by a microplate reader (BioTek, United States). Regarding colony formation experiment, 1,000 cells were seeded in cell culture plates and allowed to grow until visible colonies formed. Then we used methanol to fix clones 15 min, 1% crystal violet to stain clones 20 min, and counted the number of clones (>50 cells).

### Transwell Migration and Wound Healing Assays

BGC823 and MKN45 cells were transfected with GLP2R siRNA for 48 h and cultured in 24-well culture plates with 8 mm pore-containing membrane inserts to measure cell migration capacity. 4 × 10^4^ cells were seeded on the upper transwell chambers in 200 µl serum-free culture medium, and 500 µl medium containing 20% FBS was added to the lower chambers. After 24 h incubation, the cells that migrated through membranes were fixed with methanol, stained with 1% crystal violet and counted under light microscope (200×). Additionally, BGC823 and MKN45 cells were cultured in 24-well-plates and scraped with a 10-μl pipette tip. The cells were cultured in RPMI 1640 medium without FBS. Images of wounds were captured at 0, 24, and 48 h, the area of wounds was quantified by ImageJ software (40×).

### Statistical Analysis

All data of this study were statistically analyzed by R software 3.6.1 and Prism 9.0. The data were analyzed by two-tailed Student’s *t*-test and one-way analysis of variance (ANOVA). The difference was considered statistically significant when the *p*-value was less than 0.05.

## Results

### Primary Genetic Alterations and Statistical Analysis in STAD

**FIGURE 1:**
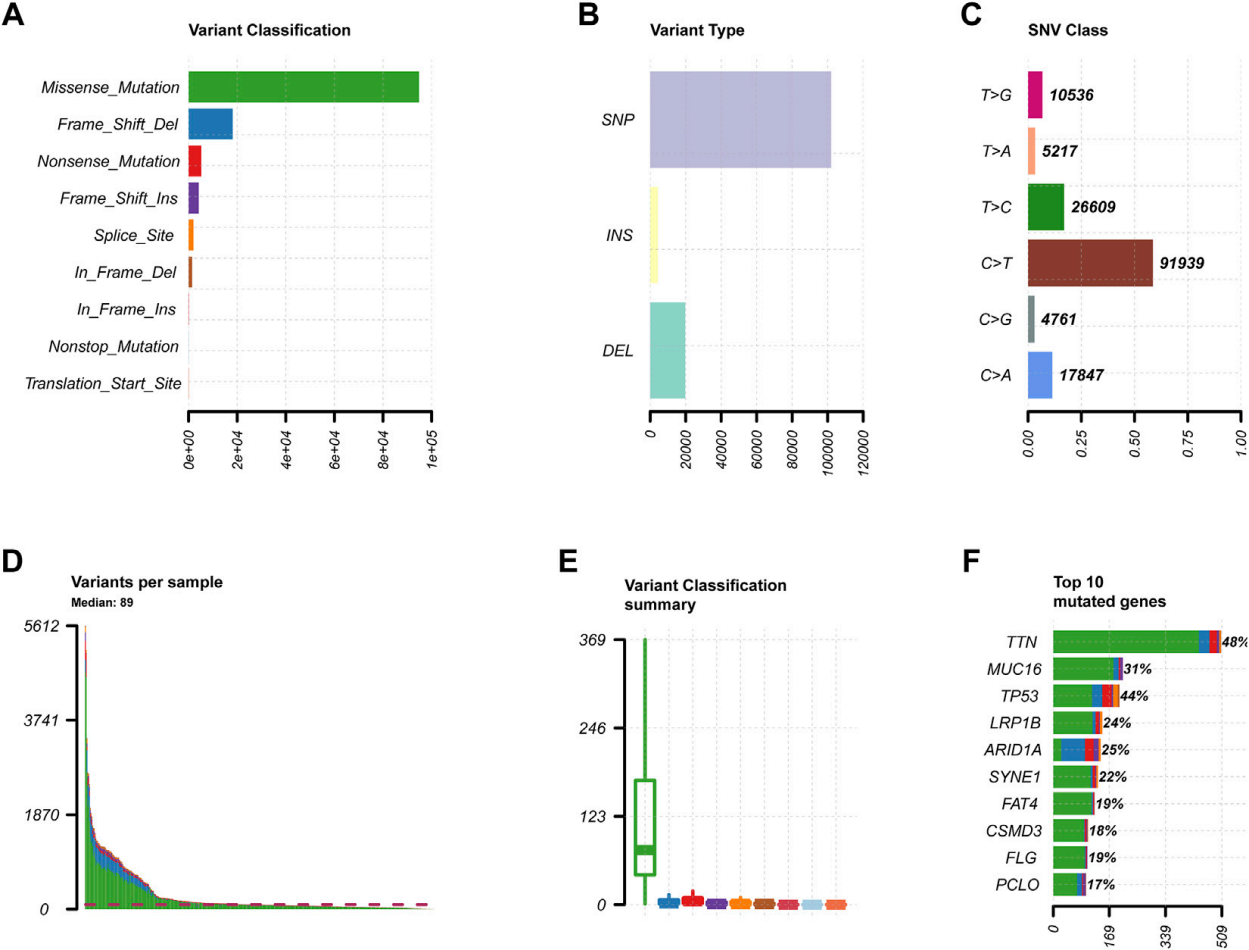
Primary genetic changes in STAD. **(A,B)** Gene variant categories in STAD. **(C)** The SNV classification of STAD. **(D,E)** The STAD samples’ mutations. **(F)** STAD mutation profile and first 10 mutant genes.

**FIGURE 2:**
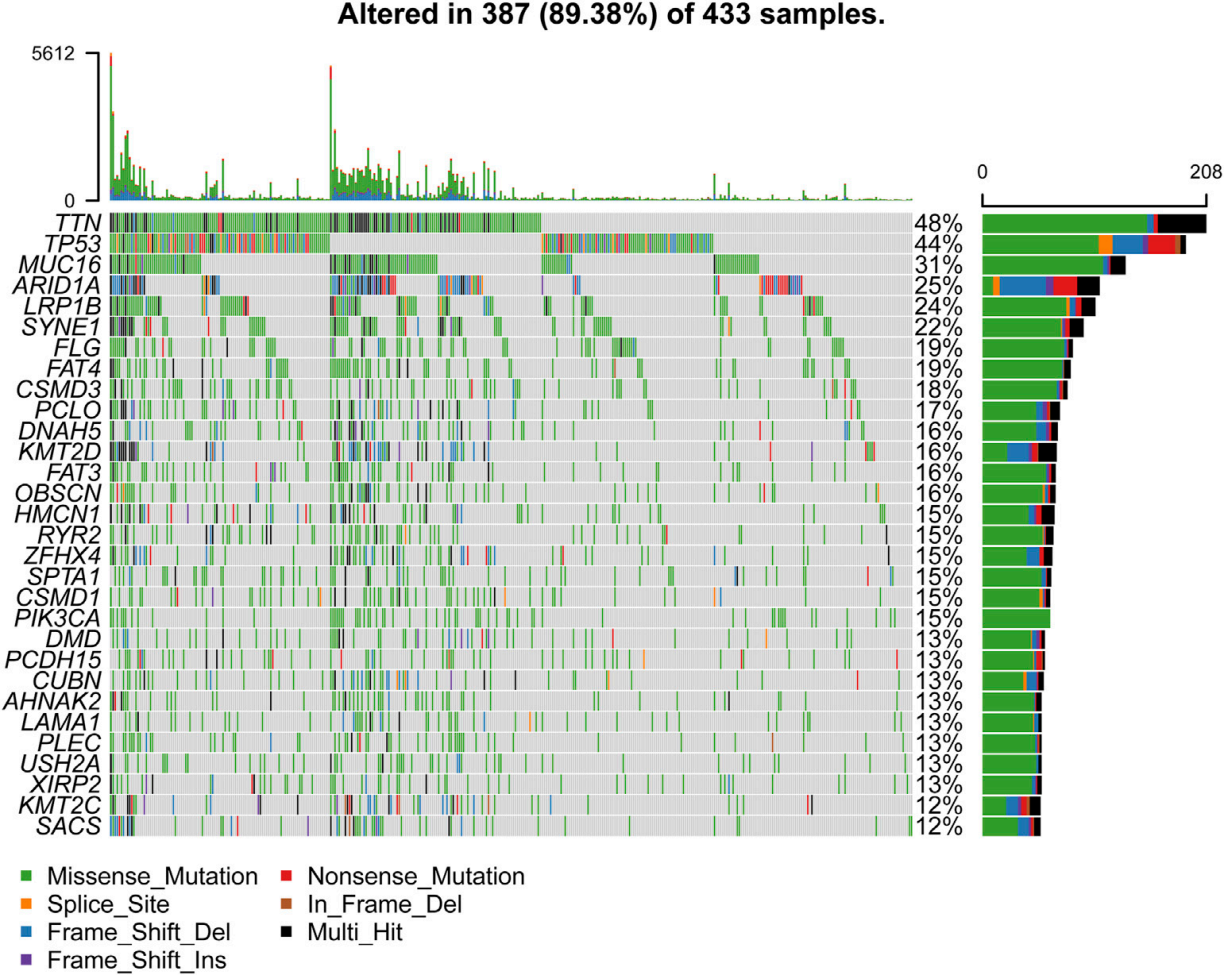
The waterfall diagram of STAD samples.

**FIGURE 3:**
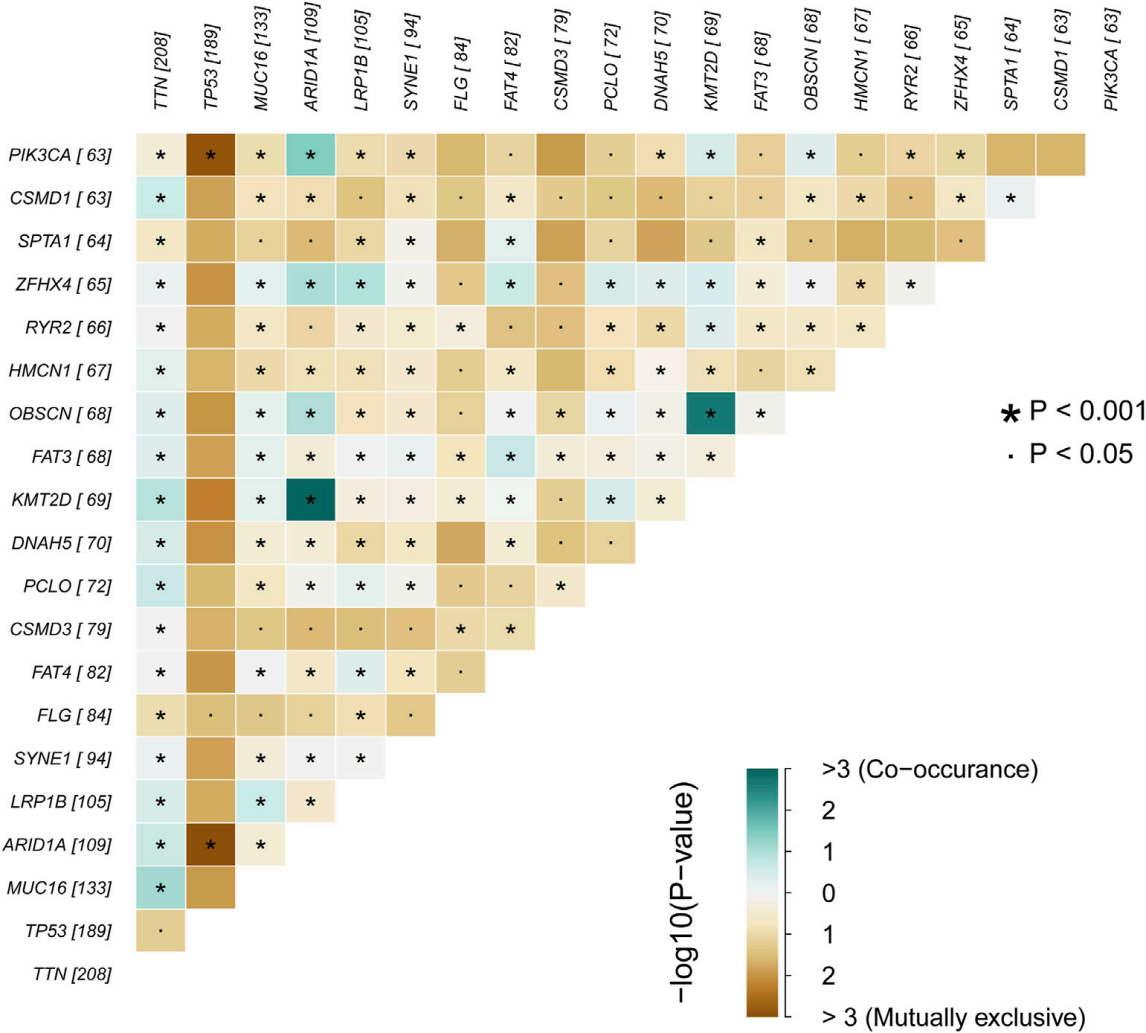
The correlation graph of STAD samples.

We obtained the clinical information and whole-exome sequencing results of STAD patients from the TCGA database (Table 1). Maftools were applied to summarize the mutation data. We further categorized mutations based on the variant effect predictor, among which the frequency of missense mutations is highest (Figure 1A). Among all mutation types, the occurrence frequency of SNP is the highest (Figure 1B). Similarly, the most frequent type of SNV in STAD is C > T transversion (Figure 1C). Figure D shows the mutation of each sample, which is most relevant to TMB (Figure 1D). Figure E also depicts the mutations of the samples (Figure 1E). The first 10 mutated genes are TTN, MUC16, TP53, LRP1B, ARID1A, SYNE1, FAT4, CSMD3, FLG, and PCLO (Figure 1F). The waterfall diagram graphically shows the gene mutations of the samples (Figure 2). The correlation graph visualizes the correlation between two gene mutations; for example, mutations of PIK3CA and ARID1A co-occur, while mutations of PIK3CA and TP53 are mutually exclusive (Figure 3).

**TABLE 1:** Clinical characteristics of STAD patients in the TCGA database.

Characteristics		Total	%
All		338	100.00
Age (y)	≥65	189	55.92
	<65	149	44.08
Gender	Male	216	63.91
	Female	122	36.09
Grade	G1	7	2.07
	G2	116	34.32
	G3	215	63.61
	G4	0	0.00
Stage	I	42	12.43
	II	112	33.14
	III	153	45.27
	IV	31	9.17
T stage	T1	15	4.44
	T2	72	21.30
	T3	167	49.41
	T4	84	24.85
M stage	M0	319	94.38
	M1	19	5.62
N stage	N0	104	30.77
	N1	95	28.11
	N2	71	21.01
	N3	68	20.12

TMB was calculated by dividing the non-synonymous protein-coding variance by the genome’s overall sequence length. Based on the median TMB, we classified STAD patients into two groups: high TMB and low TMB (Supplementary File S1). The survival rate of patients in the high TMB group is higher than that of patients in the low TMB group, according to Kaplan-Meier survival analysis (Supplementary Figure S1A). The results show that in STAD, TMB is favorably connected with age and negatively correlated with stage, T, and N stages; female patients have higher TMB than male patients. TMB in female patients is higher than in male individuals (Supplementary Figures S1B–H). Between the high and low TMB groups, 816 DEGs were found (Supplementary File S2).

### GSEA Enrichment and Immune Infiltration Analysis

**FIGURE 4:**
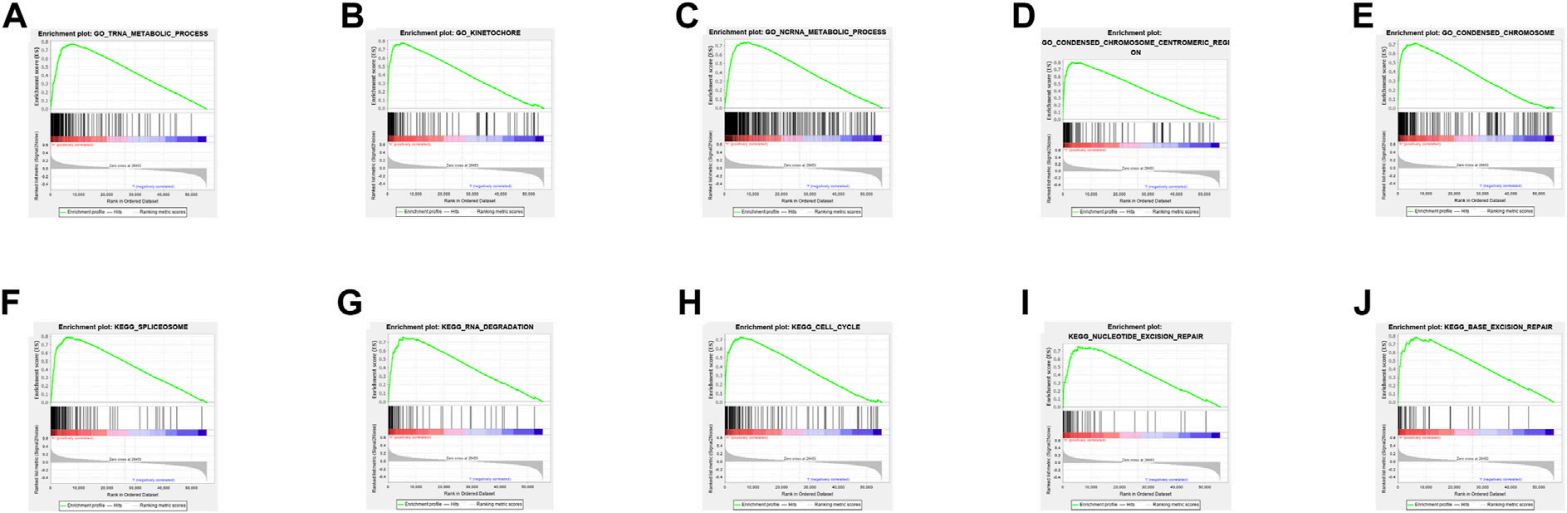
Results of GSEA enrichment. **(A–E)** GO enrichment results. **(F–J)** KEGG enrichment results.

The top 5 GO keywords (Figures 4A–E) and KEGG pathways (Figures 4F–J) with the highest significant enrichment in the high TMB group (*p* < 0.05) were obtained using GSEA enrichment analysis comparing the high and low TMB groups. These KEGG pathways and GO keywords are mostly related to genetic material metabolism and DNA repair.

**FIGURE 5:**
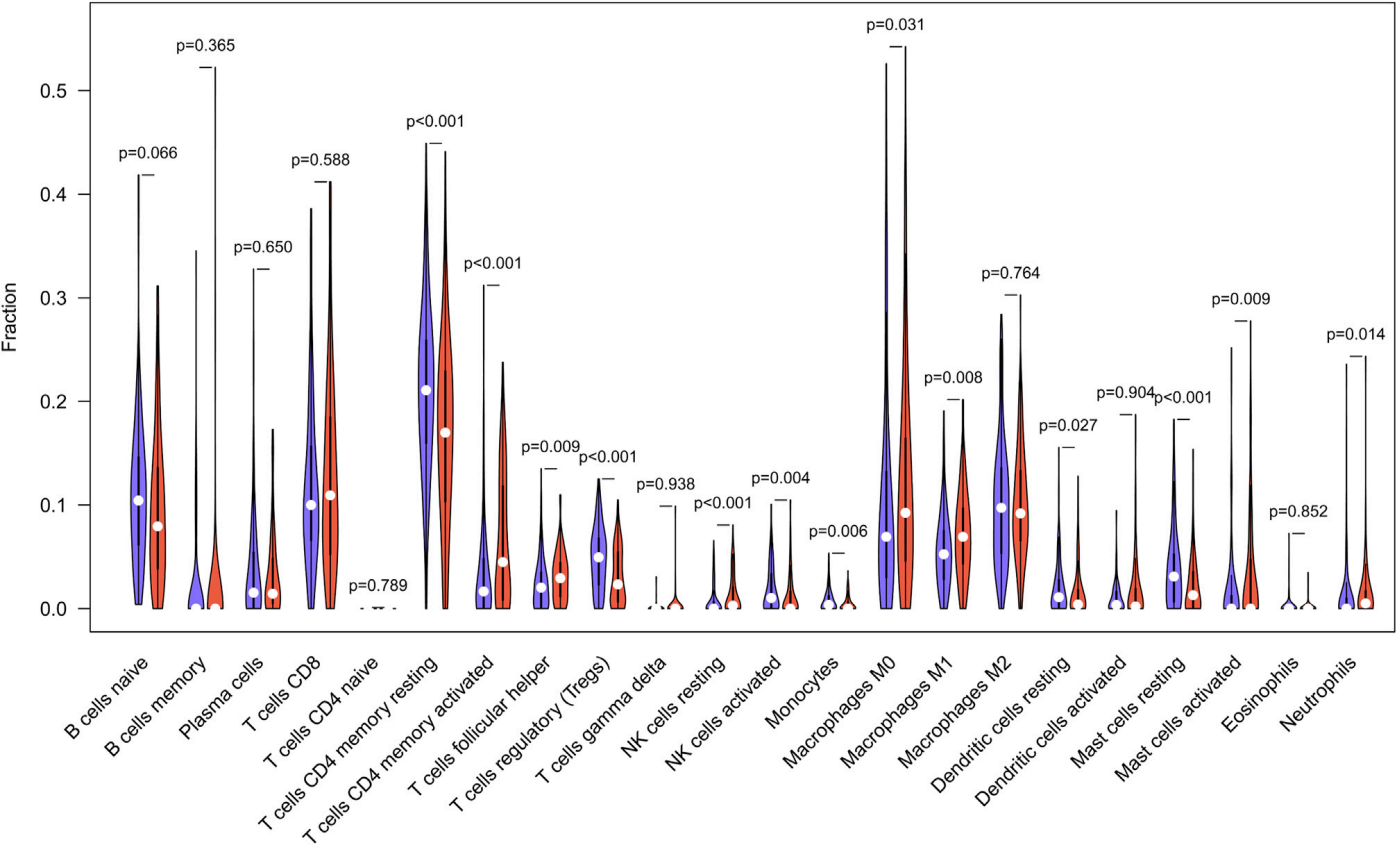
TMB and immune markers in STAD were found to be correlated. In high (red violin part) and low (blue violin part) TMB groups, there are 22 types of adaptive and innate immune cells (by wilcox test).

According to studies, the higher the TMB in tumors, the more neoantigens are produced, making tumors more immunogenic (Rizvi et al., 2015). As a result, we investigated the link between TMB and immune markers in STAD. Using the CIBERSORT method, we determined the fraction of invading immune cells. The inference of immune cell types generated by CIBERSORT was relatively reliable at the *p* < 0.05 threshold. These findings demonstrated that high TMB tumors included large proportions of follicular helper T cells, activated memory CD4+ T-cells, M1 macrophages, M0 macrophages, and Neutrophils. Resting memory CD4+ T cells, regulatory T cells, monocytes, resting dendritic cells, and resting mast cells were all higher in the low TMB group (Figure 5).

### Construction and Validations of Prognostic Model

From the overlap of immune-related genes and prior DEGs, differentially expressed immune genes were derived (Supplementary File S3). One-time random grouping was used to create the training (Supplementary File S4) and internal validation groups (Supplementary File S5). Based on univariate COX analysis (*p* < 0.01) for the training group (Supplementary File S6), we applied the LASSO algorithm to deal with the 21 survival-associated immune genes. Following the stepwise multivariate Cox regression analysis, four immune genes were able to develop a predictive model. The riskScore for each patient is calculated as follows: 0.001*APOD +0.005*APOH +0.039*INHA +0.499*GLP2R (Table 2). Each model gene is considered to with high risk attribute.

**TABLE 2:** Multivariate COX regression analysis results of model genes.

Id	Coef	HR	HR.95 L	HR.95H	*p* Value
APOD	0.001461317	1.001462385	0.999632616	1.003295504	0.117313047
APOH	0.005260581	1.005274442	1.002123723	1.008435067	0.001021469
INHA	0.039468481	1.04025771	1.008357221	1.073167407	0.013003103
GLP2R	0.499454853	1.64782272	1.065644695	2.548053521	0.024713044

**FIGURE 6:**
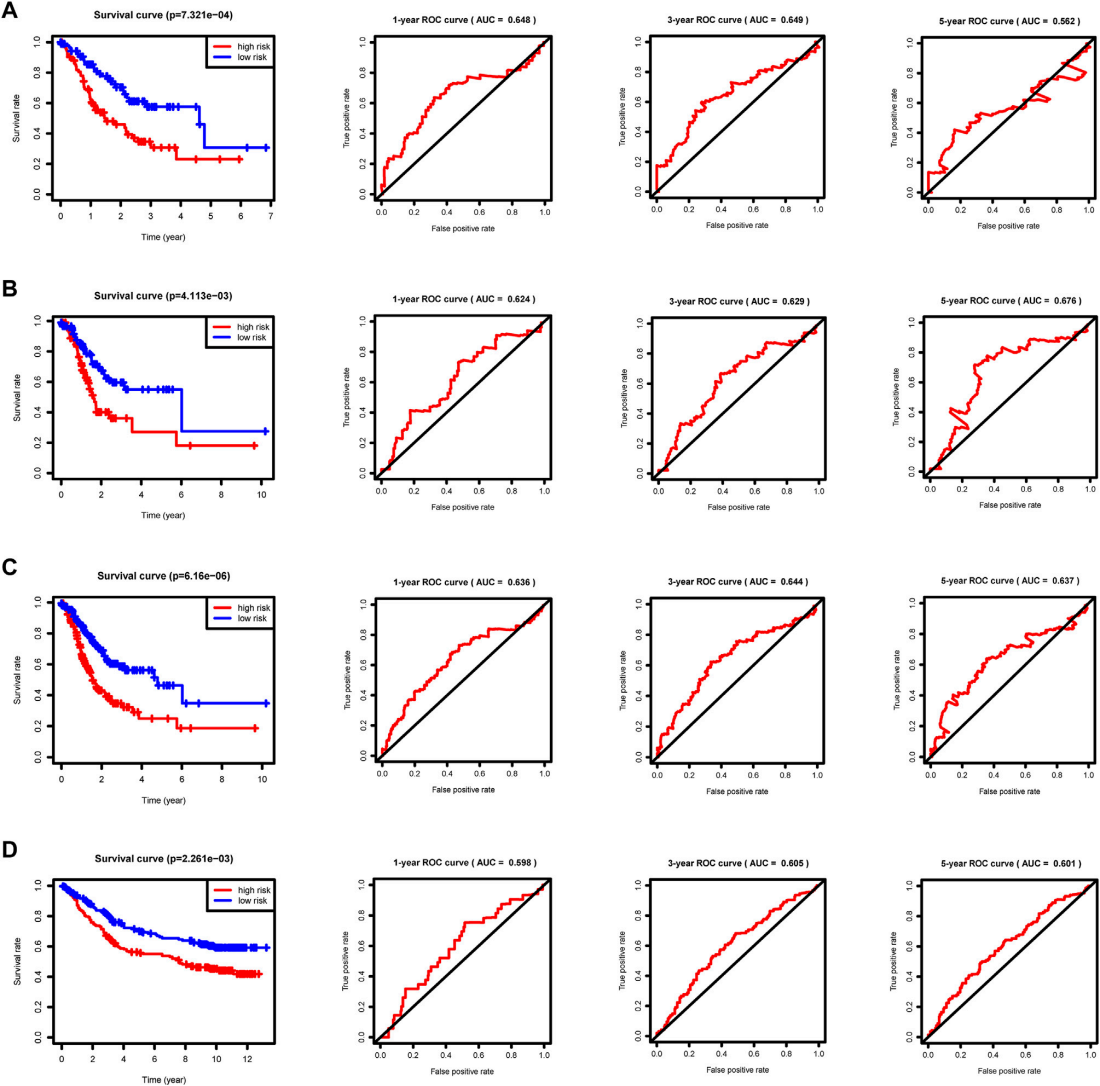
Survival curves and ROC curves. *p* < 0.05 reveals significant survival differences. The ROC curves show the model prediction efficiency. **(A)** Training group. **(B)** Internal validation group. **(C)** Total group. **(D)** External validation group GSE84433.

We split the patients in the training, internal validation, total, and external validation groups into high and low risk groups (Supplementary Files S7, S8, S9, S10) for later survival analysis after computing their riskScores. The survival curves between the high and low risk groups in the training, internal validation, total, and external validation groups are notably different, and the low risk group’s survival rate is significantly greater than the high risk group’s. The accuracy of the prognostic model we developed is dependable, according to the area under the curve (AUC) values of ROC curves (Figure 6). Ultimately, univariate and multivariate prognostic studies (*p* < 0.05) show that the riskScore derived from the model is a prognostic factor that is independent of other factors (Supplementary Figure S2).

**FIGURE 7:**
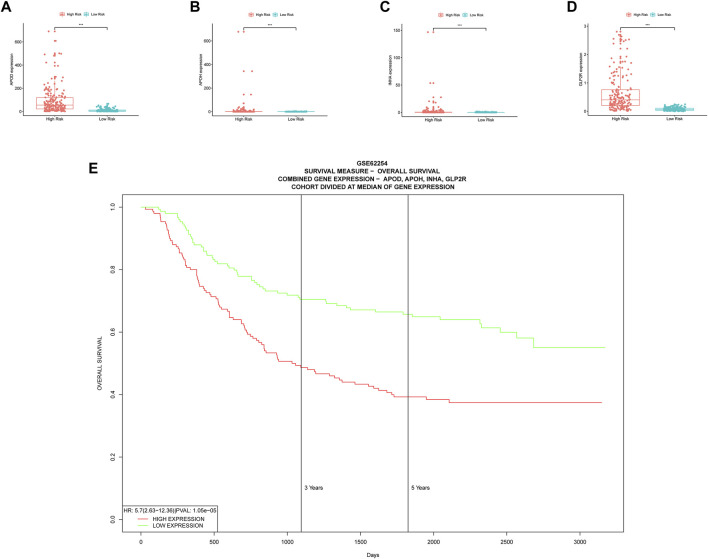
Multiple validations of model genes. **(A–D)** Boxplots display the differential expression for model genes. *, *p* < 0.05. **, *p* < 0.01. ***, *p* < 0.001. **(E)** Multiple gene survival validation, from the PROGgeneV2 online tool.

Boxplots of model genes were created to compute the expression level differences between the high and low risk groups (Figures 7A–D). The high-risk group has significantly higher expression of all model genes, which validates the earlier inference that all model genes belong to high risk factors. Furthermore, incorporating all model genes in a multiple gene survival study successfully verifies the efficiency of the prognostic model (Figure 7E). A dynamic nomograph App online (https://u20131050.shinyapps.io/STAD-TMB-Dynamic_nomogram/) was successfully designed to quickly compute patient survival and support clinical decision making in order to improve the clinical translational implications of our study. We take low-risk group sample TCGA-VQ-AA6J and high-risk group sample TCGA-D7-A6F0 as examples to show the calculation results of dynamic nomograph App, which shows that the prediction results are relatively accurate (Supplementary Figure S3).

### Alteration of Model Genes and Immunity

**FIGURE 8:**
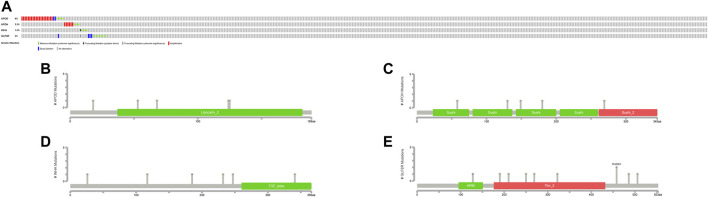
Mutation profiles of model genes. **(A)** The mutation summary of genetic alteration. Each color refers to corresponding alteration attribute. **(B–E)** Gene domain mutation. Each color refers to corresponding domain.

The cBioportal website provided an overall view of genetic change (Figure 8A) as well as domain mutation plots (Figures 8B–E) for the model genes. These model genes have extremely low mutation frequencies.

**FIGURE 9:**
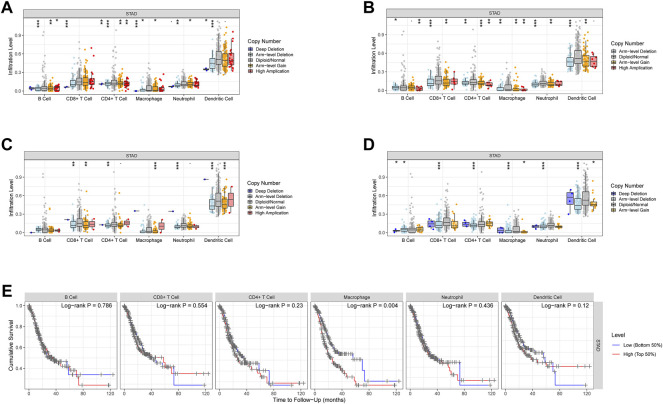
CNV of the model genes and immune cells in STAD. **(A–D)** The effect of CNV of these model genes on the penetration level difference of immune cells. **(E)** The correlation between the recruitment of immune cells and the survival of STAD patients.

According to the TIMER database, CNV of these model genes has little effect on immune cell infiltration level differences (Figures 9A–D), and only Macrophage cell infiltration has a substantial impact on STAD patients’ survival (Figure 9E).

### Visualization of Mutation Data and ssGSEA Analysis

**FIGURE 10:**
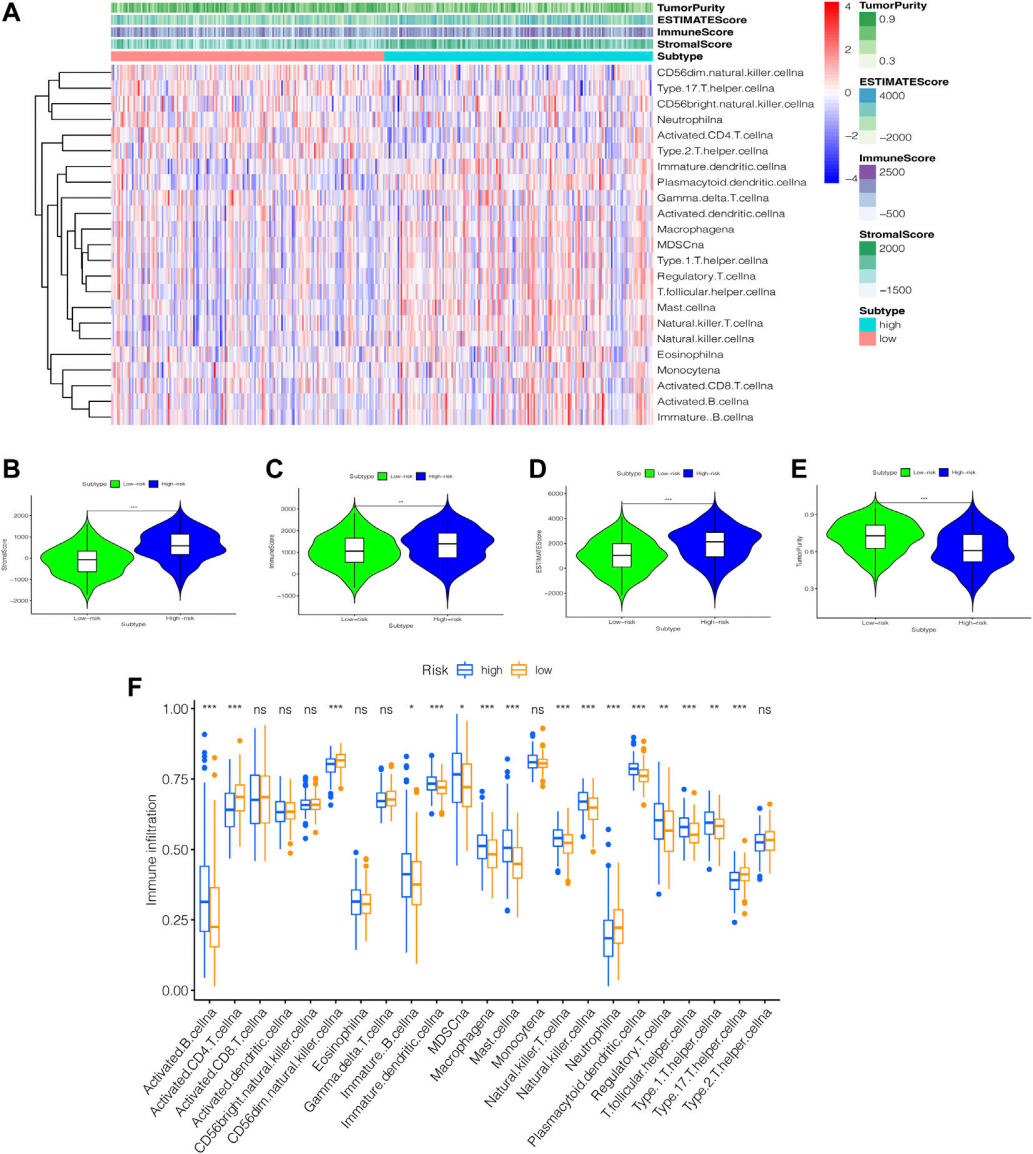
An examination of the immune status of the tumor microenvironment. **(A)** heatmap. The sample name is represented by the abscissa, and the immune gene set is represented by the ordinate. The upper section contains tumor microenvironment scores, in which Stromal Score, Immune Score, and ESTIMATE Score decrease as risk low, implying that the content of corresponding cells decreases. Tumor purity is diametrically opposed to them. **(B–E)** Violin Plot. The statistical relationship between the degree of risk and each tumor microenvironmental parameter is investigated. **(F)** box plot. Immune cell subset differences in high- and low-risk groups. *, *p* < 0.05. **, *p* < 0.01. ***, *p* < 0.001. ns, *p* > 0.05.

We created a heatmap (Figure 10A) and violin plots (Figures 10B–E) of the tumor microenvironment by analyzing the correlation between risk categorization and tumor microenvironment. In conclusion, high-risk group had higher StromalScore, ImmuneScore, and ESTIMATEScore than low-risk group, while the low-risk group had a higher score of TumorPurity. To investigate the relationship between the risk score and immune status, we used the ssGSEA method to quantify 23 immune cell subsets, discovered that the infiltration of 13 immune cell subsets was significantly higher in the high-risk group than in the low-risk group (*p* < 0.05). (Figure 10F).

### Efficacy Prediction of Immunotherapy and Model Comparison

**FIGURE 11:**
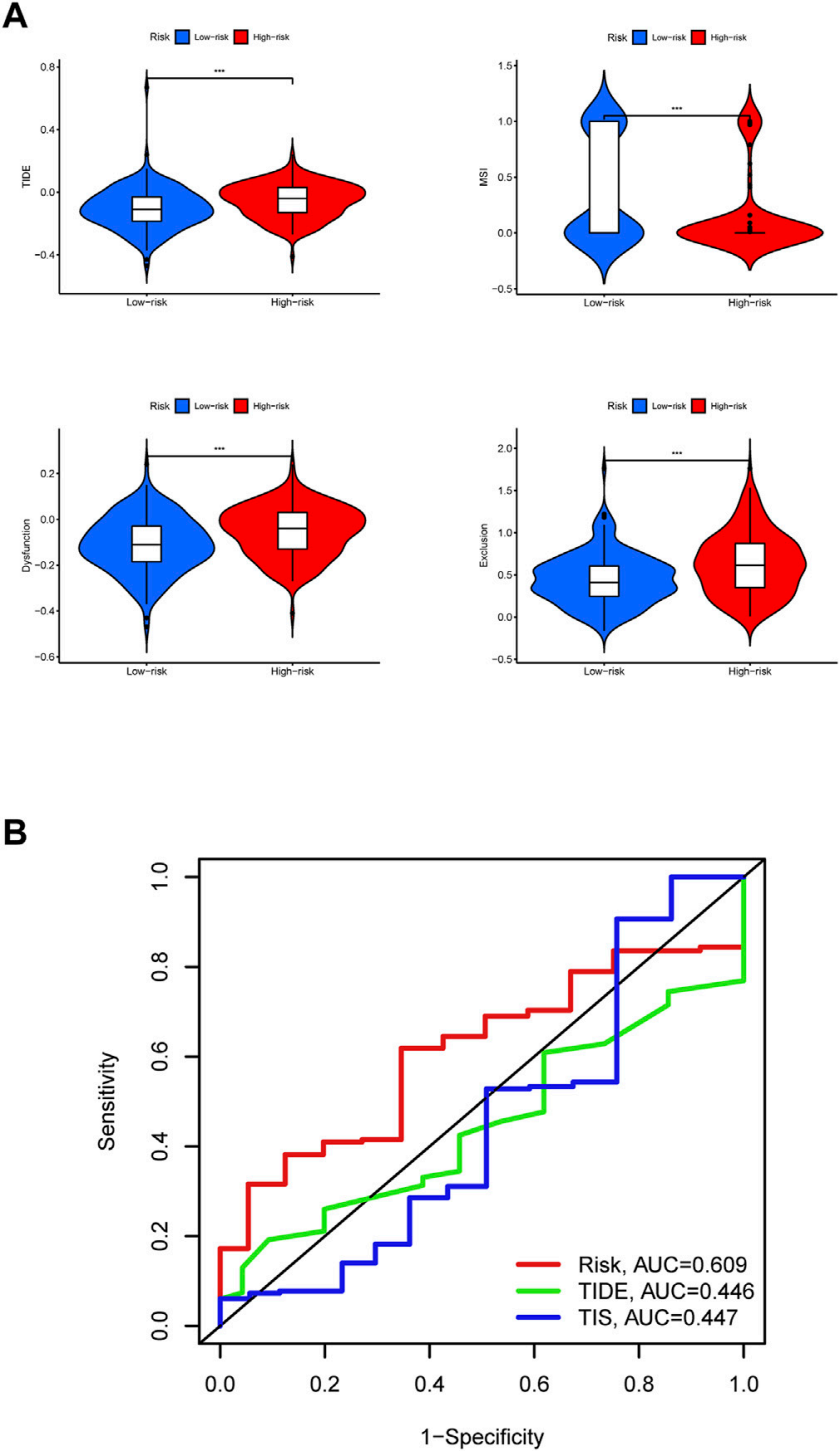
Efficacy prediction of immunotherapy and model comparison. **(A)** Violin plots. The comparison of immunotherapy biomarkers in high and low risk categories. *, *p* < 0.05. **, *p* < 0.01. ***, *p* < 0.001. **(B)** 5-years ROC curves.

The high risk group had a higher TIDE score, lower MSI score, higher Dysfunction score, and higher Exclusion score, indicating that the high risk group of STAD has increased immune escape potential, resulting in poor immunotherapy efficacy (Figure 11A).

In the end, it can be judged from the 5-years ROC curves that the prognostic model constructed by us has the largest AUC value, so its prognostic prediction efficiency is higher than TIDE score and TIS score (Figure 11B). The above results show that the group with high expression of GLP2R has the highest risk score and the worst prognosis.

### Drug Sensitivity Testing

**FIGURE 12:**
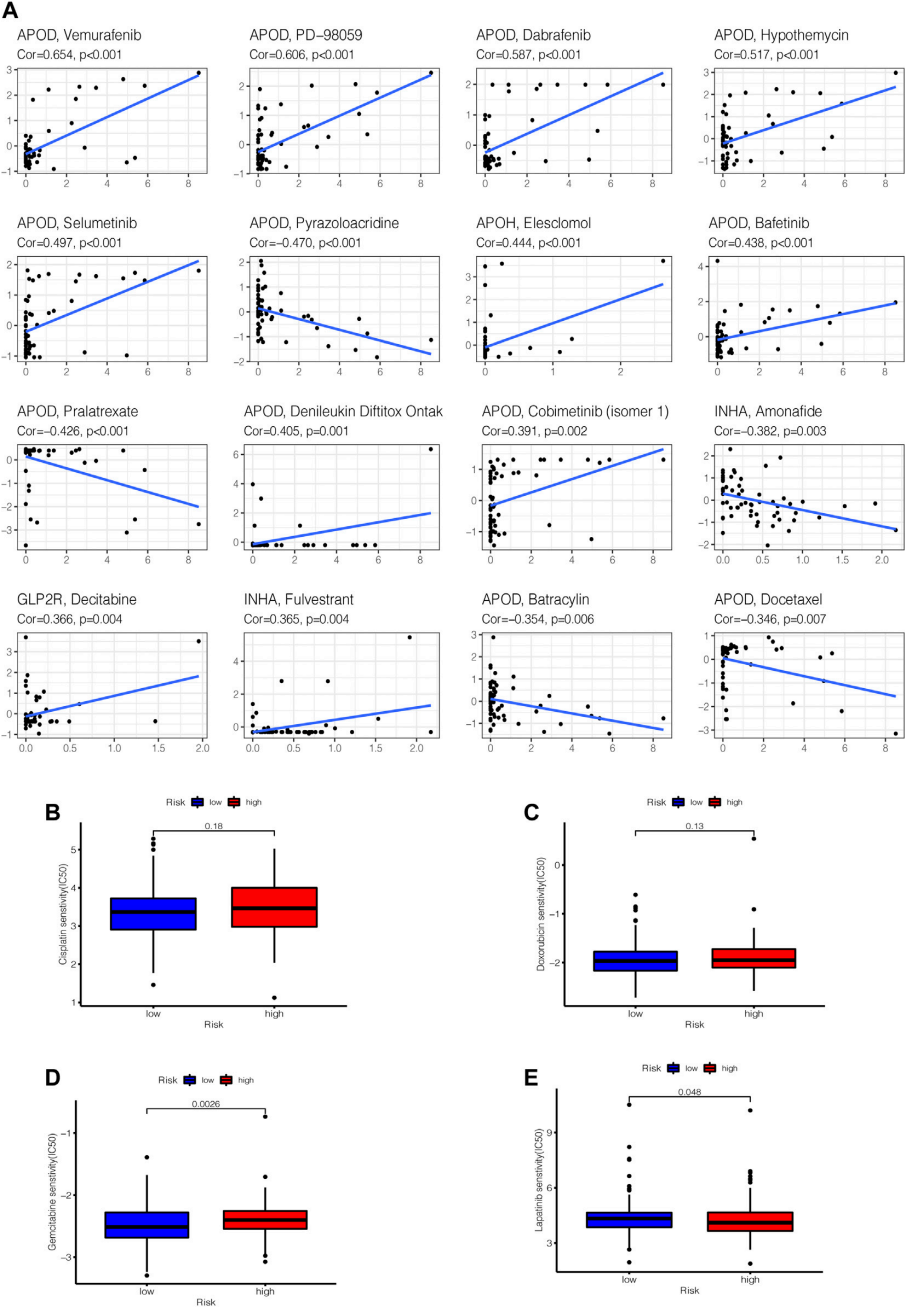
Gene–drug sensitivity analysis. **(A)** based on the CellMiner database, the top 16 strongest correlations are shown. **(B–E)** Used the “pRRophetic” R package to compare differences in drug susceptibility between high and low risk groups, the drug sensitivity of **(B)** Cisplatin, **(C)** Doxorubicin, **(D)** Gemcitabine, **(E)** Lapatinib in high and low risk groups, respectively displayed.

By performing a separate drug sensitivity analysis on model genes in the prognostic model, we were able to identify the top 16 drugs with the most statistically significant differences. APOD expression was found to be positively related to the sensitivity of vemurafenib, pd-98059, dabrafenib, hypothemycin, selumetinib, bafetinib, denileukin diftitox ontak and cobimetinib (isomer 1), it indicates that the higher level of APOD expression, the greater sensitivity to the aforementioned drugs, but APOD has a negative correlation with pyrazoloacridine, batracylin, docetaxel and pralatrexate. APOH expression was revealed to be highly linked to the sensitivity of elesclomol, INHA expression was discovered to be strongly tied to the sensitivity of fulvestrant, but inversely correlated with the sensitivity of amonafide. Furthermore, the higher the expression of GLP2R in STAD patients, the greater the patient’s sensitivity to decitabine (Figure 12A). To further improve the clinical value of tumor mutation burden-related prognostic model for the treatment of stomach cancer. We analyzed the commonly used drugs in the clinical treatment of gastric cancer, which include cisplatin, doxorubicin, gemcitabine, lapatinib, and gemcitabine was noticed to be more sensitive in the high-risk group than in the low-risk group (*p* < 0.001), whereas lapatinib is the opposite (Figures 12B–E).

### Downregulation of GLP2R Inhibits STAD Cell Proliferation and Migration

**FIGURE 13:**
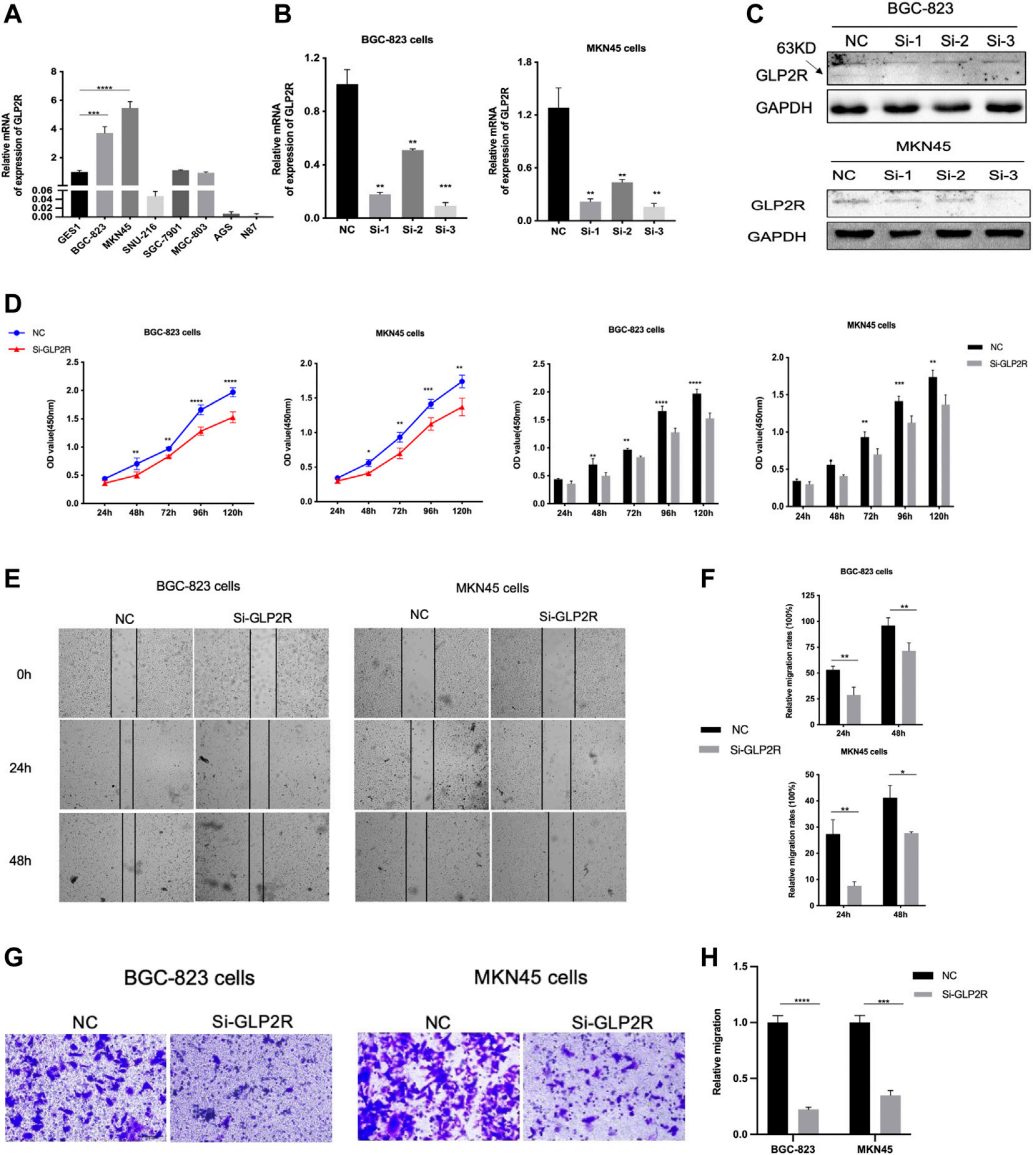
Knockdown of GLP2R inhibits proliferation, migration of gastric cancer cells. **(A)** The relative expression level of GLP2R in the GES1, BGC-823, MKN45, SNU-216, SGC-7901, MGC-803, AGS and N87 cell lines detected by RT-PCR. **(B)** The transfection efficiency of si-GLP2R in the BGC-823 and MKN45 cell lines detected by RT-PCR. **(C)** Western blot analysis confirmed that the expression of GLP2R was inhibited by GLP2R siRNA administration. **(D)** The CCK-8 assay was used to detect the effect of si-GLP2R on the proliferation of BGC-823 and MKN45 cell lines. **(E)** Representative images of the wound healing assay. **(F)** Statistical analysis of the wound healing assay results after Knockdown of GLP2R. **(G)** Representative images of the transwell assay. **(H)** Statistical analysis of the transwell assay results after Knockdown of GLP2R in the BGC-823 and MKN45 cell lines.*p < 0.05, **p < 0.01, and ***p < 0.001.

To evaluate the specific role of GLP2R in gastric cancer, the relative mRNA expression level of GLP2R in the eight gastric cancer cell lines (GES1, BGC-823, MKN45, SNU-216, SGC-7901, MGC-803, AGS and N87) was analyzed. The expression level of GLP2R in the BGC-823, MKN45 cell lines was higher than that in the other cell lines (Figure 13A). We first evaluated the transfection efficiency of the cells by qRT-PCR and Western blot, found that the relative expression level of GLP2R was significantly lower after siRNA 3 transfection (Figures 13B,C). To further confirm the role of GLP2R in proliferation, We performed CCK-8 assays to detect the effect of GLP2R knockdown. After GLP2R silencing, BGC-823, MKN45 cell proliferation significantly decreased compared to control cells (Figure 13D). Colony formation assay also indicated that GLP2R silencing significantly suppressed the growth of BGC-823 cell (Supplementary Figures S4A–B). Transwell and wound healing assays were performed to detect migration, Our results showed that the migration rates of BGC-823, MKN45 cells transfected with siRNA were significantly lower than that of the control-transfected cells (Figures 13E–H). These data suggest that GLP2R knockdown repressed the proliferative and migratory abilities of BGC-823, MKN45 cells.

## Discussion

Cancer is an inherited disease, and the mechanisms underlying involve the accumulation of harmful somatic mutations, which leads to a phenotypic consequence. Mutations are caused by faulty repair after DNA replication (especially meiosis) errors or DNA damage (such as exposure to radiation or carcinogens). Mutational processes contribute to different cancers, and the distribution of the rates of different mutational processes in various cancer types also varies (Martincorena and Campbell, 2015). High gene alterations make the tumor more immunogenic, making it a target for immune cell activation and susceptible to programmed cell death -1 (PD-1) immunocheckpoint inhibitors. TMB has been linked to a positive response to checkpoint inhibitor treatment (Snyder et al., 2014; Van Allen et al., 2015). In patients with advanced gastric cancer, a recent study found that pembrolizumab immunotherapy has good antitumor activity and low toxicity (Amatatsu et al., 2018). Due to the generation of immunogenic neoantigens, TMB, an emerging biomarker for response to immunotherapy, has also been a possible predictor of clinical benefits from immunocheckpoint inhibitors across several tumor types (Chalmers et al., 2017; Gong et al., 2019).

Higher TMB, as calculated from the complete exome, is related with greater immunotherapy responsiveness in patients with melanoma or non-small cell lung cancer, according to some studies (Chalmers et al., 2017). TMB levels beyond a certain threshold are more likely to create neoantigens in the tumor microenvironment, boosting antigenic presentation and enticing T cells to infiltrate tumors (Seto et al., 2019). A series of stromal cells, including vascular cells, fibroblasts, and inflammatory cells, come into being the tumor microenvironment (Greten and Grivennikov, 2019). More and more evidence suggests that the tumor microenvironment is important in the beginning and progression of STAD. Meanwhile, tumor-infiltrating lymphocytes have been shown to be effective in predicting GC patient prognosis, demonstrating that immune-related protective and hazardous variables in the GC microenvironment can become prognostic predictors (Yang et al., 2019).

TMB appears to have a considerable impact on the prognosis of STAD, in a study that included TGCA data from 375 gastric cancer patients, 15.7% of the patients had high TMB, and screening 632 up-regulated genes and 979 down-regulated genes were selected (Bai et al., 2020)**,** further pathway analysis showed that patients in the high TMB group were associated with high activated CD4+ memory T cells, follicular helper T cells, quiescent NK cells, M0 and M1 macrophages, and neutrophils Cell infiltration.

TMB has been reported in many articles in tumor patients, as a novel immunotherapy marker, TMB levels are linked to immune cell infiltration in the tumor microenvironment, the higher the TMB, the better the outcome (Li et al., 2016; Kelly, 2017; Wei et al., 2021).

The clinical data of 63 patients with advanced stomach cancer who received immunotherapy were analyzed, assessed whether TMB is associated with response to immunotherapy, the results showed that high TMB was associated with the effectiveness of ICI treatment and chemotherapy, and no progression in the high TMB group extended survival, so TMB may serve as a predictive biomarker for patients with advanced gastric cancer treated with ICI material that aids clinical decision-making (Kim et al., 2020).

We acquired 816 DEGs by comparing the low and high TMB groups. We can identify a close association between genetic material metabolism, DNA repair, and TMB based on the GSEA enrichment data. TMB deficiency impairs genetic material metabolism and is linked to the DNA repair mechanism, an enzyme system that prevents changes in genetic material, as seen in non-small cell lung cancer (Chae et al., 2019). According to our findings, those with a high TMB had more activated memory CD4+ T-cells, follicular helper T cells, M0 macrophages, M1 macrophages, and Neutrophils. High TMB encourages the invasion of these immune cells, as evidenced by studies in different malignancies (Zhang et al., 2019; Wu et al., 2020; Xu et al., 2020). These findings suggested that TMB can alter the features of immune cell infiltration, and that TMB levels beyond a certain threshold can attract immune effector cells. The relative balance between immunosuppressive and anti-tumor immune cells is one of the methods by which tumor cells retain immune-mediated dormancy (Mittal et al., 2014). Activated memory CD4+ T-cells and follicular helper T cells are archetypal anti-tumor immune cells, as we all know. Follicular helper T cells are a subpopulation of CD4 T cells that support B cells in the germinal center of lymphatic tissue. Classically activated M1 macrophages may be implicated in the early removal stage of immunoediting driven by CD8 cytotoxic T lymphocytes and interferons, where they destroy tumor cells and cause tissue death (Quaranta and Schmid, 2019). TMB and the immune microenvironment have a very close interaction, as can be observed. In the growth of malignancies, the interaction of these immune cells is critical.

According to the results of the ssGSEA analysis, the degree of TMB in STAD patients is positively correlated with the level of immune infiltration. According to GSEA enrichment analysis, the risk was positively correlated with StromalScore, ImmuneScore, and ESTIMATEScore, but negatively coupled to TumorPurity, and the higher risk, the stronger immunosuppressive activity. This finding could explain why STAD patients with higher risk levels have a lower survival rate.

In our study, which also indicates that the risk score of the model composed of APOD, APOH, INHA, and GLP2R. At the same time, we demonstrates that increased GLP2R expression is attached to an increase in cancer cell sensitivity to decitabine. Decitabine is a chemotherapeutic pyrimidine nucleoside analogue used for the treatment of myelodysplastic syndromes by inducing DNA hypomethylation and corresponding alterations in gene expression (https://go.drugbank.com/drugs/DB01262). Therefore, we guess that the model gene can be used as an evaluation index of drug efficacy.

It's almost probable that the model genes we discovered are high-risk genes, and that the prognostic model we built is accurate. Through bioinformatics analysis, we found that GLP2R has the highest risk coefficient. And GLP2R was reported to be associated with gastrointestinal cancer in previous studies (Lu et al., 2019; Qiu et al., 2020). Therefore, in order to investigate whether it can be used as a prognostic indicator, we performed in gastric cancer cell lines (BGC823, MKN45) and found that knockdown of GLP2R can significantly inhibit the proliferation and migration of gastric cancer cell lines.

Although there is little study on the significance of these model genes in STAD, they are essential in other malignancies, therefore further mechanism studies are needed. For example, APOD can be employed as a therapeutic tool to promote tumor cell death as a prognostic marker for numerous cancer types (Søiland et al., 2009; Bajo-Grañeras et al., 2013). Similarly, INHA induces tumor angiogenesis and promotes tumor metastasis as a poor survival predictor of various cancer types, and is a potential target for anti-angiogenesis therapy and genetic engineering therapy (Singh et al., 2018; Yoon et al., 2018).

The results of mutation study show that our model genes have low mutation frequency. Perhaps, on the one hand, the model genes in our study do not rely on structural and functional alterations to influence STAD prognosis; on the other hand, they generate high risk effect in STAD as a result of quantitative changes. The judgment could also be explained by the fact that the CNV of these model genes has no effect on the difference in immune cell infiltration levels. Only Macrophage cells, out of the six types of immune cells, can alter STAD patients’ survival. Combined with previous violin plot of immune cell infiltration, to a large extent, Macrophages M0, M1 rather than M2, may play a vitalrole in the prognosis of STAD. At present, studies on the role of Macrophage cells in STAD are still lacking. Therefore, this is a direction worth exploring.

## Conclusion

In conclusion, our results imply that STAD patients with a high TMB have a better prognosis. We identified DEGs between high and low TMB groups using STAD samples from the TCGA database, and investigated the relationship between immune cell infiltration features and TMB. In addition, the immune prognostic model was built using a range of bioinformatics methodologies, with several validations. Our study’s clinical translational importance was further strengthened by the establishment of the dynamic nomograph App online and immunotherapy prediction based on the prognostic model. Finally, GLP2R may be expected to be a potential target for gastric cancer.

## Data Availability

The datasets presented in this study can be found in online repositories. The names of the repository/repositories and accession number(s) can be found in the article/Supplementary Material.

## Abbreviations

AUC, Area under the curve; CNV, Copy number variation; DEGs, Differentially expressed genes; DNA, Deoxyribonucleic acid; GEO, Gene Expression Omnibus; GC, Gastric cancer; GSEA, Gene Set Enrichment Analysis; GO, Gene Ontology; ICI, Immune checkpoint inhibitor; EMT, Epithelial-mesenchymal transition; FDR, False discovery rate; KEGG, Kyoto Encyclopedia of Genes and Genomes; LASSO, Least absolute shrinkage and selection operator; MSI, Microsatellite instability; PD-1, Programmed cell death -1; ROC, Receiver Operating Characteristic; STAD, Stomach adenocarcinoma; TCGA, The Cancer Genome Atlas; TIMER, Tumor Immune Estimation Resource; TIDE, Tumor immune dysfunction and exclusion; TIS, Tumor inflammation signature; TMB, Tumor mutation burden.
