# Supplementary figures and images for: Development of Tumor Mutation Burden-Related Prognostic Model and Novel Biomarker Identification in Stomach Adenocarcinoma
Source: Front Cell Dev Biol. 2022 Mar 23;10:790920. doi: 10.3389/fcell.2022.790920 (PMC8983817; doi:10.3389/fcell.2022.790920)

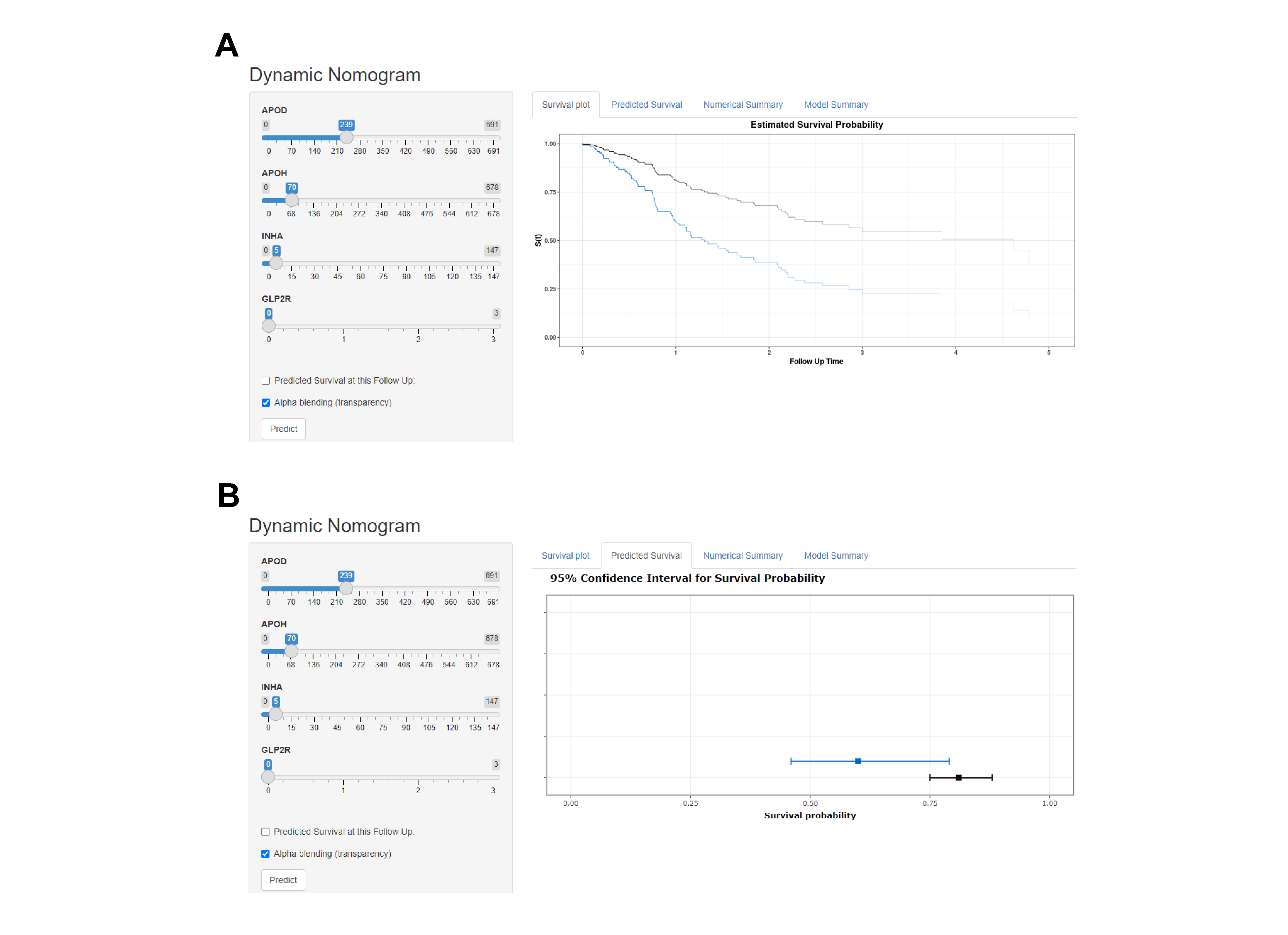

Supplement: Supplementary file 1 [file Image3.TIF]

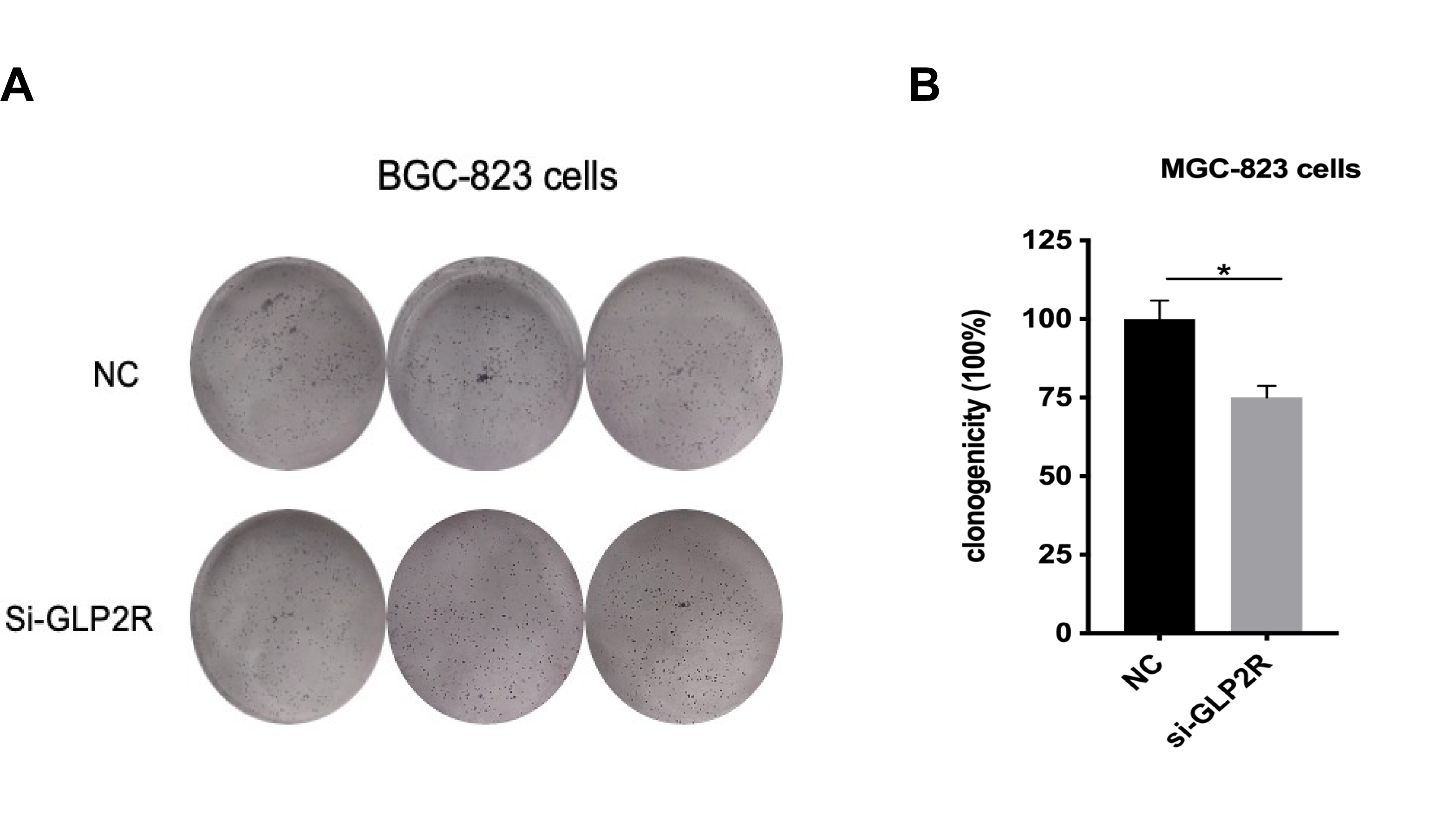

Supplement: Supplementary file 2 [file Image4.tif]

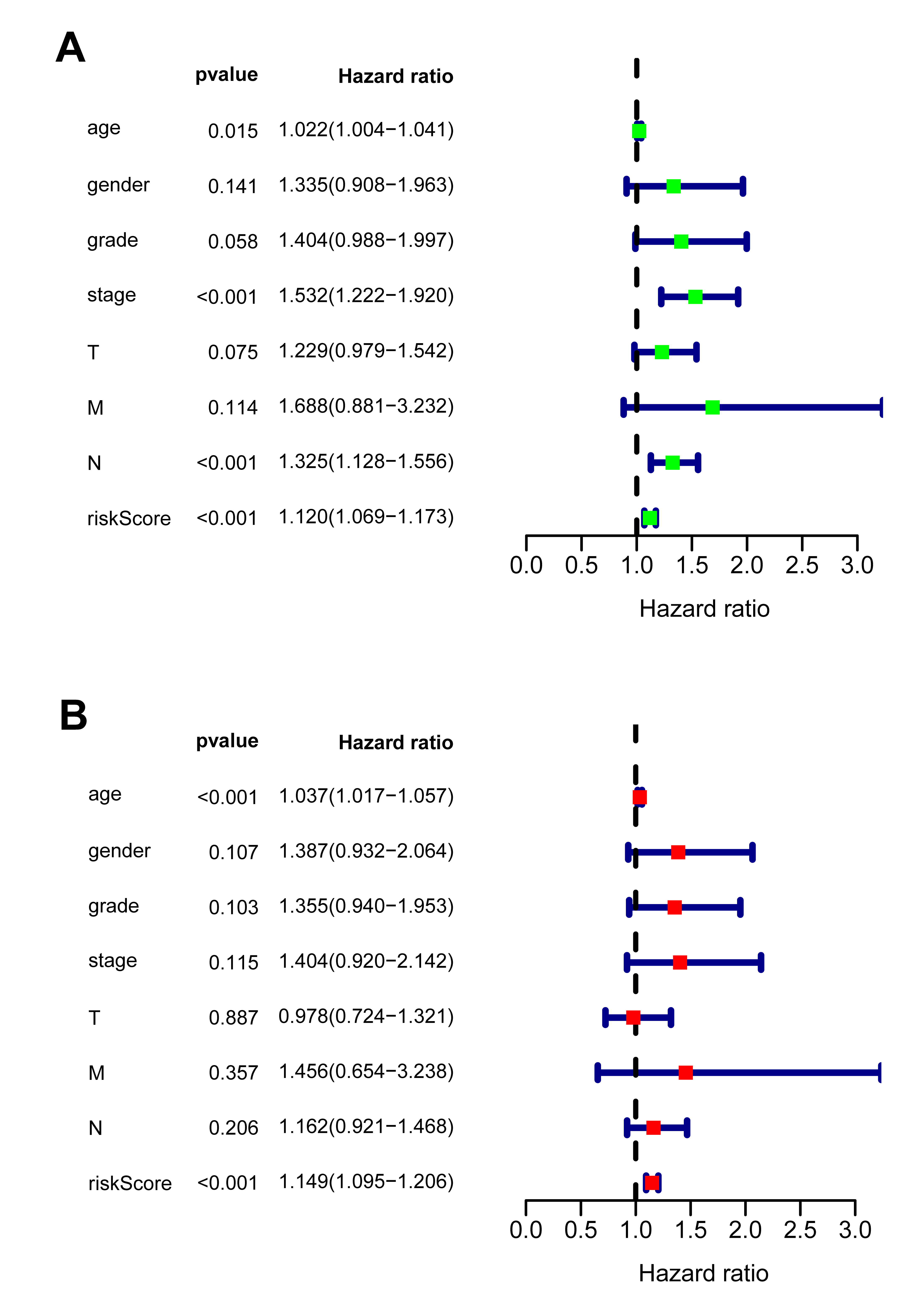

Supplement: Supplementary file 4 [file Image2.TIF]

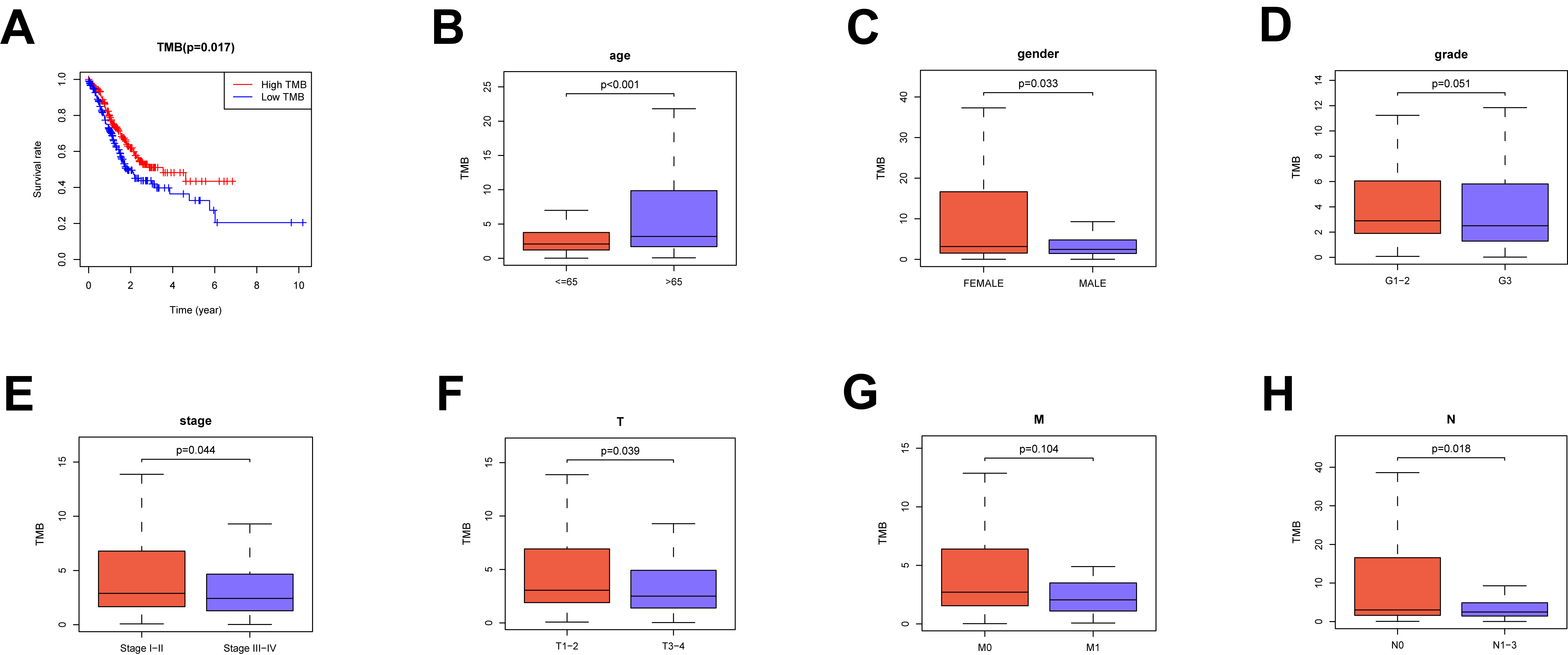

Supplement: Supplementary file 5 [file Image1.TIF]
